# Effect of Caffeine Injection on Orthodontic Tooth Movement in Rats: An Experimental Study on Rats

**DOI:** 10.1155/2022/7204806

**Published:** 2022-01-25

**Authors:** Amin Golshah, Khaled Omidi, Nafiseh Nikkerdar, Fatemeh Ghorbani

**Affiliations:** ^1^Department of Orthodontic, School of Dentistry, Kermanshah University of Medical Sciences, Kermanshah, Iran; ^**2**^ Student Research Committee Kermanshah University of Medical Sciences, Kermanshah, Iran; ^3^Department of Maxillofacial Radiology, School of Dentistry, Kermanshah University of Medical Sciences, Kermanshah, Iran; ^4^Medical Biology Research Center, Health Technology Institute, Kermanshah University of Medical Sciences, Kermanshah, Iran

## Abstract

**Objectives:**

Several studies have investigated the effects of different medications on orthodontic tooth movement (OTM). This study assessed the effect of caffeine injection on OTM in rats.

**Materials and Methods:**

Thirty-five male Wistar rats were randomly divided into five groups. Their first molars and central incisors were attached with a nickel-titanium closed coil spring with 50 g load. The rats in the three experimental groups received 25, 50, and 75 mg/kg caffeine intraperitoneally for 21 days. The negative control group did not receive any injection and did not undergo orthodontic treatment. The positive control group underwent orthodontic treatment and received 0.9% NaCl (saline) injection. After 21 days, all rats were sacrificed by chloroform inhalation, and the maxilla was resected. The mean number of Howship's lacunae, blood vessels, osteoclasts, and root resorption lacunae was histologically measured. The bone volume-to-total volume ratio (BV/TV) in maxillary molars was calculated by microcomputed tomography (micro-CT) to quantify bone loss.

**Results:**

The amount of OTM and the number of osteoclasts, blood vessels, and Howship's lacunae significantly increased in rats under caffeine therapy, while the number of root resorption lacunae did not increase. Lower BV/TV in the caffeine groups was in accord with the increased count of osteoclasts.

**Conclusion:**

Caffeine injection can significantly increase OTM in rats.

## 1. Introduction

Orthodontic treatment has gained wide public acceptance in the recent years. It not only corrects dental anomalies but also improves the esthetic appearance of the face. Moreover, orthodontic treatment corrects temporomandibular joint disorders due to malocclusion [1]. Orthodontic tooth movement (OTM) is induced by mechanical stimuli and enhanced by the periodontal ligament and alveolar bone remodeling [2, 3]. Mechanical loads induce the local cellular responses in the periodontal ligament and alveolar bone and enable OTM [4, 5]. Bone remodeling is a dynamic process that occurs following the interactions of bone-forming osteoblasts and bone-resorbing osteoclasts [2, 6]. Orthodontic forces can increase the intracellular secondary messengers, which are critical for osteoblastic differentiation and OTM [7]. Thus, theoretically, any factor affecting the osteoclastic function can affect the OTM as well [8, 9]. To shorten the treatment course, orthodontists have long been in search of methods to accelerate OTM. Also, orthodontists should have adequate information about the medications that can change the bone physiology because they can probably affect the treatment course as well [10, 11]. Recent evidence confirmed the effect of several systemic factors on OTM. Some medications have an adverse effect on this process and can decelerate the treatment course, while some others can shorten the duration of treatment [12, 13].

Caffeine (1,3,7-trimethylxanthine) is among the most common central nervous system stimulants used in drinks, foods, and medications [14, 15]. Caffeine has several activities at the cellular and molecular levels, affecting phosphodiesterase, adenosine receptors, prostaglandins, and proinflammatory mediators [4]. Caffeine can lead to osteoclastogenesis through the COX-2/prostaglandin E2 (PGE2) pathway [16]. Consumption of caffeine-containing products may affect the bone metabolism [17]. Since caffeine has various effects on the bone metabolism, it may affect the OTM as well, particularly if consumed on a daily basis [10].

The reports regarding the effects of caffeine on bone metabolism, bone density, and bone regeneration have been controversial. Some authors reported that caffeine caused osteoporosis and periodontal disease [15, 18, 19], while some others found no significant correlation between bone loss, bone density, or disease conditions with caffeine consumption [20, 21]. Also, it has been demonstrated that caffeine can positively affect the process of mineralization and enhance the mechanical properties of the skeletal tissue if consumed at certain times [4].

Evidence shows that the genome of rats and humans is highly similar such that approximately 90% of the human genes have direct orthologs in rats [22]. Thus, we hypothesized that daily injection of caffeine may enhance OTM. Previous studies on this topic mainly evaluated the effects of oral administration of caffeine, and studies regarding the effects of caffeine injection are lacking [10, 15, 16]. Thus, this study aimed to assess the biological and histological effects of the intraperitoneal injection of caffeine on OTM in rats.

## 2. Materials and Methods

### 2.1. Animal Model and Treatment Methods

The study was approved by the ethics committee of Kermanshah University of Medical Sciences (IR.KUMS.REC.1397.724) and conducted in accordance with the ARRIVE guidelines [23]. This animal study evaluated 35 male Wistar rats weighing 200–250 g (all rats were purchased from Kermanshah University of Medical Sciences). The rats were kept in transparent plastic cages under standard laboratory conditions at a constant temperature of 24-25°C, 55% humidity, and 12 h dark/12 h light cycles for one week prior to the experiment for the purpose of acclimation [24]. The rats were fed soft food to minimize the appliance movement after installation. The orthodontic appliance was installed for all rats except for the negative control group as explained elsewhere [25]. Injections were made at 1, 3, 5, 7, 9, 11, 13, 15, 17, 19, and 21 days after appliance placement. The rats were randomized into five groups (*n* = 7) [26, 27] as follows.

Group 1 received intraperitoneal injection of 25 mg/kg caffeine at the aforementioned time points for 3 weeks simultaneously with orthodontic treatment.

The rats in group 2 received intraperitoneal injection of 50 mg/kg caffeine at the aforementioned time points for 3 weeks simultaneously with orthodontic treatment.

The rats in group 3 received intraperitoneal injection of 75 mg/kg caffeine at the aforementioned time points for 3 weeks simultaneously with orthodontic treatment.

The negative control group did not receive any injection and did not undergo orthodontic treatment. Caffeine was purchased from Sigma-Aldrich (St. Louis, MO, USA).

The rats in the positive control group received intraperitoneal injection of 0.9% NaCl at the aforementioned time points for 3 weeks simultaneously with orthodontic treatment.

### 2.2. Placement of the Orthodontic Appliance

The rats were anesthetized with 10% ketamine hydrochloride (50 mg/kg; Alfasan, Woerden, Netherlands) and 2% xylazine (2 mg/kg; Alfasan, Woerden, Netherlands). After anesthesia induction, the vital signs of the rats were closely monitored. Also, they were rotated from side to side every couple of minutes to prevent pulmonary edema. The room temperature was also controlled. The nickel-titanium closed coil springs were installed (G&H Franklin, 6 mm, 50 g) to induce OTM. The first molars and central incisors were connected by a stainless steel wire. Next, the first molars and central incisors were etched with 37% phosphoric acid (Vivadent, USA) for 30 s, rinsed for 10 s, and dried with an air spray for 15 s. Single Bond (3M ESPE, St. Paul, MN, USA) was applied on the surface and light-cured with a LED curing unit (Woodpecker, Muenster, Germany) with a light intensity of 150 mw/cm^2^ for 10 s followed by the application of Transbond XT composite (3M ESPE, St. Paul, MN, USA).

**Figure 1:**
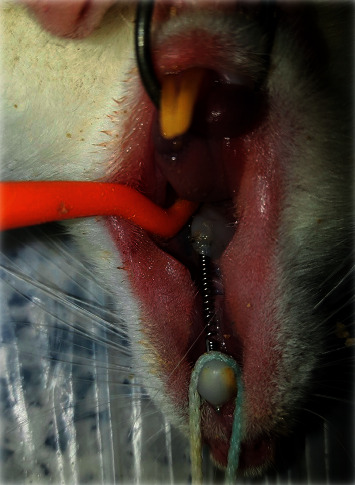
Nickel-titanium closed coil spring installed between the first molars and central incisors.

The load applied by the coil springs was 50 g [28] (Figure 1). The magnitude of load applied by the coil spring was measured by using a force meter upon installation. To protect the orthodontic appliance, the lower teeth were reduced. The rats were examined daily to ensure the presence of the coil spring at its rightful place. In case of debonding of the appliance, the rat would be excluded and replaced [29].

### 2.3. OTM

**Figure 2:**
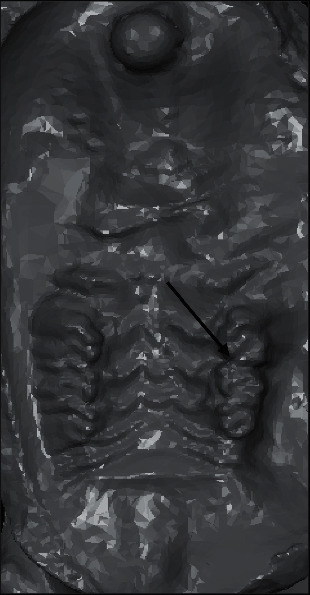
Three-dimensional scan of the cast of a rat that received caffeine simultaneously with orthodontic treatment using ABViewer software. The arrow indicates the amount of OTM.

The distance between the enamel of the most distal part of the first molar crown and mesial surface of the second molar was measured by an examiner blinded to the group allocation of rats at baseline and after 21 days. ABViewer 14 software was used for this purpose. Each measurement was repeated 3 times, and the mean of the measurements was reported as the final value. Impressions were made from the teeth at 1 and 21 days using injection silicone polyvinyl siloxane impression material (Express; 3M Dental Products, St. Paul, MN, USA). After 4 min (to allow polymerization), the impressions were checked and poured with dental stone (Elite Rock Dental Stone; Zhermack, Badia Polesine, Italy). After 24 h, the gypsum cast was removed. The casts were scanned with a 3D scanner (inEos X5; Sirona Dental Systems, Bensheim, Germany) to create 3D models in an STL format [30] (Figure 2). Next, the STL files were sent to ABViewer 14 software for the measurements.

### 2.4. Histological and Immunohistochemical Analyses

#### 2.4.1. Tissue Preparation

After 21 days, the rats were sacrificed by inhalation of chloroform in a desiccator. The maxilla was resected and sent for histological analysis. The specimens were fixed in 10% formaldehyde and decalcified in 10% formic acid for 48 h (Sigma-Aldrich; St. Louis, MO, USA). After fixation, decalcification of the tissue was performed using 12.5% ethylenediaminetetraacetic acid, and fixation was performed by using a fixator for 10 weeks. The decalcifying solution was agitated 10 times a day and refreshed twice weekly until complete decalcification occurred. Next, they were dehydrated by using ethanol and embedded in paraffin blocks. Parasagittal sections were made by using a microtome (Leica, Wetzlar, Germany) with 5 *µ*m thickness [31]. An examiner (A. H. Y) who was blinded to the group allocation of specimens performed all the histological analyses.

#### 2.4.2. Histological Assessment

Tissue specimens were stained with hematoxylin and eosin, and the slides were inspected by an experienced pathologist who was blinded to the group allocation of specimens under a light microscope (Eclipse E400, Nikon, Japan) at 100x magnification. The number of Howship's lacunae, blood vessels, osteoclasts, and root resorption lacunae was also counted in an area measuring 0.01 mm^2^. Each specimen was assessed three times, and the mean of the three measurements was recorded.

### 2.5. Microcomputed Tomography (Micro-CT) Analysis

In this study, we used an in vivo X-ray microcomputed tomography (micro-CT) scanner. LOTUS-inVivo has a cone beam microfocus X-ray source and a flat panel detector. In order to obtain best possible image quality, the X-ray tube voltage and its current were set to 60 kV and 130 *µ*A, respectively, and frame exposure time was set to 1 second by 1.4 magnification. Total scan duration was 28 minutes. Slice thicknesses of reconstructed images were set to 50 micrometers. All the protocol settings were controlled by LOTUS-inVivo-ACQ software. The acquired 3D data were reconstructed using LOTUS-inVivo-REC by a standard Feldkamp, Davis, and Kress (FDK) algorithm. Also, LOTUS-inVivo-3D was used for rendering of reconstructed images, and by adding the bone analysis plugin (BAP) inside software, we reported bone volume (BV) and total volume (TV) parameters [32].

### 2.6. Statistical Analysis

The data were analyzed using SPSS version 21 (IBM, Armonk, NY, USA) by one-way ANOVA, Tukey's test, and *t*-test.

## 3. Results

The minimum intraclass correlation coefficient was calculated to be 0.964 for all variables, indicating excellent reliability of the measurements.

### 3.1. Effect of Daily Injection of Caffeine on OTM

Figure 2 presents the descriptive data regarding OTM in the five groups. After 21 days of application of orthodontic force, a significant difference was noted among the five groups in OTM (*P* < 0.05). The amount of OTM was minimum in the negative control (*P* = 0.011) and maximum in the 75 mg/kg caffeine group (*P*= 0.458). A significant difference existed between the negative control group and all other groups in OTM (*P* < 0.05). Also, significant differences existed between the positive control and 50 mg/kg caffeine and positive control and 75 mg/kg caffeine groups (*P* < 0.05). However, the difference between the positive control and 25 mg/kg caffeine group was not significant (*P* = 0.354). Increasing the caffeine dosage significantly increased the mean amount of OTM such that the difference between the 25 and 50 mg/kg (*P* = 0.005) and 25 and 75 mg/kg (*P* < 0.05) caffeine groups was significant. However, the difference between the 50 and 75 mg/kg caffeine groups did not reach statistical significance (*P* = 0.582, Table 1).

**Figure 3:**
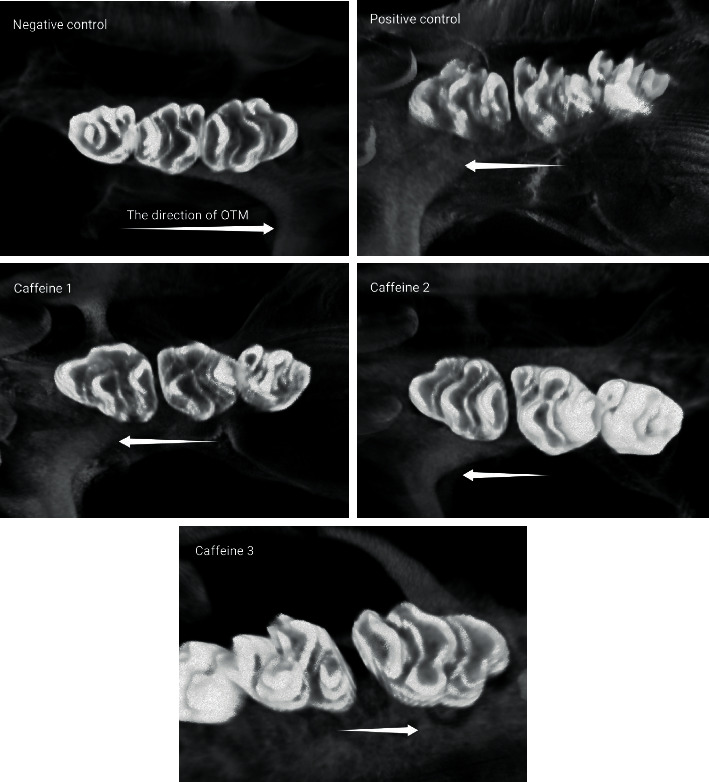
Micro-CT image (1 *µ*m = 1 × 10^−6^ m of orthodontic tooth movement (OTM) after 21 days of force application): negative control group (no OTM and no injection), positive group (OTM and saline injection), and experimental groups injected with 25, 50, and 75 mg/kg caffeine.

Figure 3 shows the micro-CT images of OTM in the five groups.

### 3.2. Histopathological Evaluation

**Figure 4:**
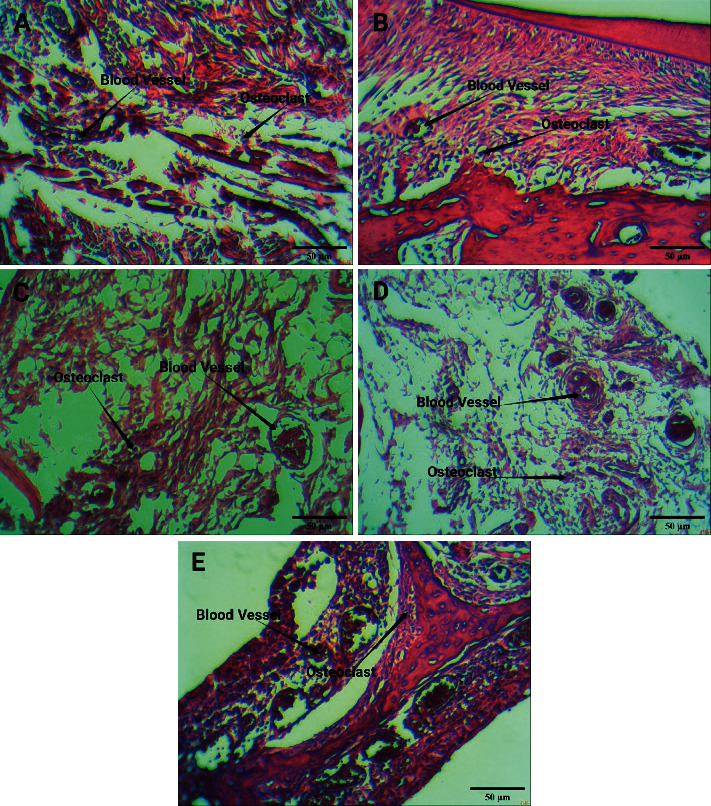
The result of HE staining. Blood vessels and osteoclasts in (a) the negative control, (b) the positive control, (c) caffeine 25 mg/kg, (d) caffeine 50 mg/kg, and (e) caffeine 75 mg/kg. Scale bar: 50 *µ*m.

Light microscopic findings of the periodontal tissue showed that, in the negative control group, the number of blood vessels and osteoclasts was histologically normal. In the positive control group, which was affected by orthodontic force, the number of blood vessels and osteoclasts increased on average, but this increase was more pronounced in the three groups of caffeine, which were simultaneously affected by orthodontic force and different doses of caffeine (Figure 4).

### 3.3. Histological Analysis

Table 2 presents the mean and standard deviation of histological variables.

### 3.4. Howship's Lacunae

At 21 days, a significant difference existed in the number of Howship's lacunae among the five groups (*P* < 0.05). The number of Howship's lacunae was maximum in the 75 mg/kg caffeine group (*P* = 4.166) and minimum in the negative control group (*P* = 0.166). Significant differences existed between the negative control and all other groups in the number of Howship's lacunae (*P* < 0.05). However, the difference was not significant between the positive control and 25 mg/kg caffeine groups (*P* = 0.540). The difference between the positive control and 50 mg/kg caffeine group was not significant either (*P* = 0.323). However, the difference was significant between the positive control and 75 mg/kg caffeine group (*P* < 0.05). The difference between the 25 and 50 mg/kg caffeine groups was not significant (*P* = 0.995), but the difference between the 25 and 75 mg/kg caffeine groups was significant (*P* = 0.014). Also, 50 and 75 mg/kg caffeine groups had a significant difference in this respect (*P* = 0.035).

### 3.5. Blood Vessels

At 21 days, a significant difference was noted in the number of blood vessels among the five groups (*P* < 0.05). The number of blood vessels was maximum in 75 mg/kg caffeine (*P* = 5.6) and minimum in the negative control group (*P* = 2.0). Also, the difference between the negative control group and the other groups was significant (*P* < 0.05). No significant difference was noted between the positive control and 25 mg/kg caffeine (*P* = 1.000) or positive control and 50 mg/kg caffeine (*P* = 0.340) groups. However, the difference between the positive control and 75 mg/kg caffeine group was significant (*P* ≤ 0.001). The difference between the 25 and 50 mg/kg caffeine groups was not significant (*P* = 0.340), but 25 and 75 mg/kg caffeine groups (*P* ≤ 0.001) and 50 and 75 mg/kg caffeine groups had significant differences in this regard (*P* = 0.002).

### 3.6. Osteoclasts

A significant difference existed among the five groups in the number of osteoclasts (*P* < 0.05). The number of osteoclasts was maximum in 75 mg/kg caffeine (*P* = 4.000) and minimum in the negative control group (*P* = 0.833). A significant difference existed between the negative control and the other groups in the number of osteoclasts (*P* < 0.05). No significant difference existed between the positive control and 25 mg/kg caffeine group (*P* = 0.139). However, the difference between the positive control and 50 mg/kg caffeine group was significant (*P* = 0.025). A significant difference was also noted between the positive control and 75 mg/kg caffeine group (*P* = 0.010). No significant difference was found between the 25 and 50 mg/kg (*P* = 0.925), 25 and 75 mg/kg (*P* = 0.742), or 50 and 75 mg/kg caffeine groups (*P* = 0.994).

### 3.7. Root Resorption Lacunae

At 21 days, a significant difference was noted in the number of root resorption lacunae among the five groups (*P* < 0.05). The number of root resorption lacunae was maximum in 50 and 75 mg/kg caffeine (*P* = 1.5) and minimum in the negative control group (*P* ≤ 0.01). No root resorption lacunae were seen in the negative control group, and this group had significant differences with all other groups in this respect (*P* < 0.05). No significant difference was noted between the positive control and 25 mg/kg caffeine (*P* = 0.672), positive control and 50 mg/kg caffeine (*P* = 0.870), positive control and 75 mg/kg caffeine (*P* = 0.672), 25 and 75 mg/kg caffeine (*P* = 0.870), or 50 and 75 mg/kg caffeine (*P* = 1.000) groups.

### 3.8. Effect of Caffeine on BV/TV Ratio

The micro-CT results revealed a significant difference in this regard among the five groups (*P* < 0.05). The BV/TV ratio was minimum in the 75 mg/kg caffeine and maximum in the negative control group. The negative control group had significant differences with the other groups in this regard (*P* < 0.05). No significant difference was found between the positive control and 25 mg/kg caffeine (*P* = 0.395) and positive control and 50 mg/kg caffeine (*P* = 0.230) groups. However, the difference between the positive control and 75 mg/kg caffeine group was significant (*P* = 0.031). Pairwise comparisons of the three caffeine groups showed no significant difference (*P* > 0.05, Table 3).

## 4. Discussion

Several studies have assessed the effects of different medications and surgical procedures on OTM. Some studies reported that certain medications or surgical approaches enhanced OTM, while some others showed deceleration of OTM by different stimuli [33–36]. Response to orthodontic treatment with respect to the speed of OTM varies among different individuals. This variation is due to the differences in bone remodeling caused by the effect of different systemic factors [37]. Some materials such as triptolide were shown to significantly decrease the amount of OTM and the root resorption area in rats. Injection of triptolides can stop OTM and decrease root resorption in rats by inhibition of osteoclastogenesis. Moreover, triptolide can positively affect osteoblastogenesis [38]. Another study showed that the level of plasma estrogen affected the speed of OTM. Estrogen inhibits osteoblastic differentiation and reinforces the apoptosis of osteoblasts and prevents alveolar resorption. This effect of estrogen decelerates the process of bone remodeling in OTM and decreases the rate of OTM as such [39].

This study assessed the effect of caffeine on OTM in rats. Rats are suitable laboratory animals to study bone remodeling and OTM in response to mechanical forces [40–42]. This study was the first to show that intraperitoneal injection of caffeine significantly increased OTM in rats. Caffeine is the most commonly used active medicinal agent worldwide, which is found in drinks such as coffee and tea, chocolate products, and some medications. Consumption of energy drinks containing high amounts of caffeine has greatly increased in the recent years [43]. An animal study showed that brewed coffee increased the level of RANKL and OPG and enhanced OTM. Thus, brewed coffee can effectively accelerate the process of orthodontic treatment [2]. Another study showed that caffeine effectively increased the level of PGE2. PGE2 serves as a stimulant for the formation of RANKL in osteoblasts and increases the number of osteoclasts and OTM [43].

The results of the present study revealed that orthodontic forces stimulated the osteoclasts and angiogenesis. Also, injection of 25, 50, and 75 mg/kg caffeine along with orthodontic force application further increased the number of osteoclasts and blood vessels. Although the difference in this respect was not significant among different doses of caffeine, caffeine injection eventually increased OTM. Increasing the caffeine dosage increased OTM. Histological assessments revealed that injection of caffeine increased the number of osteoclasts, Howship's lacunae, and blood vessels. However, it did not significantly increase the number of root resorption lacunae. The increase in the number of osteoclasts due to caffeine injection was in agreement with a previous report in this regard [16]. Micro-CT indicated a lower BV/TV ratio in the caffeine groups, which was in agreement with the increase in the number of osteoclasts. Nonetheless, several complex factors are involved in the measurement of the amount of OTM such as deformation of the appliance while chewing, mesial drift of the second molar, and cranial growth during the study period [29].

In this study, all measurements were made by the same examiner to eliminate interobserver errors, which was a strength of this study.

This study had some limitations. It was an animal study with limited sample size. The findings of animal studies should be further confirmed by clinical human studies. Ethically, a minimum acceptable number of laboratory animals should be used in animal studies, which limits the generalizability of the results. Human studies with larger sample size are required to assess the effect of caffeine on OTM over long courses of treatment.

## 5. Conclusion

This animal study indicated that caffeine can accelerate OTM in rats, and this effect was fortified by increasing the caffeine dosage. Further investigations are required to elucidate the mechanisms involved in this effect. Generalization of the results of animal studies to humans should be done with caution. Given that the results are confirmed, the skeletal effects of caffeine should also be taken into account by orthodontists in orthodontic treatment planning.

## Figures and Tables

**Table 1:** Mean and standard deviations of orthodontic tooth movement (OTM) in millimeters in the five experimental groups.

Groups	Distance of orthodontic tooth movement (OTM) at the beginning of the study (mm)		Distance of orthodontic tooth movement (OTM) at the end of 21 days (mm)		Differences between the beginning of the study and the end of the study (mm)
	Mean	SD	Mean	SD	Mean
Negative control	0.20	0.02	0.22	0.02	0.02^a^
Positive control	0.20	0.02	0.45	0.03	0.19^b^
Caffeine 25 mg/kg	0.21	0.01	0.50	0.03	0.19^b^
Caffeine 50 mg/kg	0.22	0.02	0.62	0.03	0.2^c^
Caffeine 75 mg/kg	0.21	0.02	0.67	0.03	0.2^c^

^†^Welch's one-way ANOVA test followed by Tukey's test was used. Means with the same superscript letters are not significantly different (*P* > 0.05). SD: standard deviation.

**Table 2:** The mean and standard deviation of each histologic variable in the experimental groups (mm^2^).

Group	Number of Howship's lacunae		Number of blood vessels		Number of osteoclasts		Number of root resorption lacunae	
	Mean	SD	Mean	SD	Mean	SD	Mean	SD
Negative control	0.16	0.40	2.00	0.89	0.83	0.40	0.00	0.00
Positive control	2.00	0.89	3.50	1.04	2.50	0.54	0.83	0.40
Caffeine 25 mg/kg	2.66	0.81	3.50	0.54	3.50	0.83	1.16	0.40
Caffeine 50 mg/kg	2.83	0.75	4.16	0.98	3.83	1 .16	1.50	0.54
Caffeine 75 mg/kg	4.16	0.75	5.66	0.51	4.00	0.89	1.50	0.54
*P* value^‡^	<0.001		<0.001		<0.001		<0.001	

^‡^One-way ANOVA test followed by Tukey's test was used. Means with the same superscript letters are not significantly different (*P* > 0.05). SD: standard deviation.

**Table 3:** Mean BV/TV ratios in the study groups.

Bone volume-to-total volume ratio (BV/TV, %)		Groups
SD	Mean	
3.6	74.2	Negative control
4.8	67.7	Positive control
2.8	64.0	Caffeine 1
3.4	63.2	Caffeine 2
4.2	61.2	Caffeine 3
	<0.001	*P* value^‡^

^‡^One-way ANOVA test followed by Tukey's test was used. Means with the same superscript letters are not significantly different (*P* > 0.05).

## Data Availability

No data were used to support this study.
